# Factors influencing procalcitonin in the cerebrospinal fluid of patients after neurosurgery and its diagnostic value for intracranial infection

**DOI:** 10.1186/s12883-023-03339-8

**Published:** 2023-08-01

**Authors:** Huajun Wang, Chengjie Zhou, Ye Fu

**Affiliations:** Department of Intensive Care Unit, the Affiliated People’s Hospital of Ningbo University, 251 East Baizhang Road, Ningbo City, Zhejiang Province People’s Republic of China

**Keywords:** Intracranial, PCT, Serum, Cerebrospinal fluid, Influencing factors

## Abstract

**Objective:**

This study aimed to investigate the factors influencing Procalcitonin (PCT) in the cerebrospinal fluid (CSF) of patients with high fever and suspected intracranial infection after neurosurgery and its clinical application value.

**Methods:**

Between February 2021 and August 2022, CSF and serum samples were collected via lumbar puncture from patients with high fever and suspected intracranial infection in the Intensive Care Unit(ICU) of our hospital. Multivariate logistic regression analysis was performed to analyze the factors influencing elevated PCT in CSF. The diagnostic efficacy of each index was assessed using receiver operating characteristic (ROC) curves.

**Results:**

A total of 183 CSF samples were collected, of which 148 had increased PCT levels, including 73 cases of intracranial infection and 75 cases in the case‒control group. Multivariate logistic regression analysis showed that intracranial infection [OR = 0.117, 95% CI: 0.025–0.559; *p* < 0.01] and hemorrhagic CSF [OR = 0.162, 95% CI: 0.029–0.916; *p* < 0.04] were factors influencing CSF PCT, while trauma [OR = 3.43, 95% CI: 0.76–15.45; *p* < 0.12], epileptic seizure [OR = 0.00, 95% CI: 0.00; p < 0], age [OR = 1.02, 95% CI: 0.98–1.52; *p* < 0.32] and Glasgow Coma Scale (GCS) score [OR = 1.03, 95% CI: 0.78–1.32; *p* < 0.83] did not influence CSF PCT. The CSF PCT and serum PCT levels in the intracranial infection group and the case‒control group were 0.13 (0.11, 0.25) ng/ml and 0.14 (0.07, 0.25) ng/ml and 0.14 (0.08,0.32) ng/ml and 0.23 (0.13,0.48)ng/ml, respectively, with no statistically significant difference. The median values of CSF lactate in the intracranial infection group and the case‒control group were 6.45 (4.475, 8.325) mmol/l and 3.2 (2.02, 4.200) mmol/l, respectively, with a statistically significant difference between the groups.The areas under the ROC curve of CSF PCT, serum PCT,CSF lactate, CSF PCT combined with lactate were 0.59, 0.63, 0.82,and 0.83,respectively.

**Conclusion:**

Intracranial infection and hemorrhagic CSF are influencing factors for elevated CSF PCT following neurosurgery. It should be noted that the diagnostic value of intracranial infection by CSF PCT elevated alone is limited, but the combination it with other indicators can help improve diagnostic efficacy.

## Introduction

Intracranial infection is a common complication following neurosurgery, with an incidence of 1.5–19.4% [1, 2]. Diagnostic criteria for intracranial infection include elevated temperature, clinical symptoms and laboratory results, however, a positive cerebrospinal fluid (CSF) culture is considered the gold standard. Nonetheless, various factors can affect CSF examination after neurosurgery, leading to a low positive rate in CSF cultures. Early diagnosis is crucial for the treatment of central nervous system (CNS) infections, yet there is a lack of sensitive and effective indicators for diagnosing intracranial infection following neurosurgery.

Procalcitonin (PCT) is an inflammatory index that increases significantly during severe infections. It is highly sensitive and not influenced by other factors. The International Sepsis Conference has designated PCT as a diagnostic indicator of sepsis, and it is widely utilized in the diagnosis and treating of bacterial infections [3, 4]. Recent studies have observed a rise in serum and CSF PCT during intracranial infection [5–8]. Our previous study also suggested that when the PCT in the CSF surpass serum PCT, it can serve as a diagnostic indicator for intracranial infections [9]. However, other studies have shown that the increase in CSF PCT is nonspecific since it is influenced by serum PCT when the blood–brain barrier (BBB) is compromised after neurosurgery [10–12]. Multiple organ dysfunction syndrome, pancreatitis, paraquat poisoning, among other noninfectious conditions, have been shown to elevate serum PCT levels [13, 14]. Additionally, CSF PCT has been observed to rise in nonintracranial infections. Gautam-Goyal et al. also discovered that CSF PCT was increased in patients with subarachnoid hemorrhage [15]. Consequently, these findings indicated that increased CSF PCT are nonspecific, and its diagnostic value following neurosurgery remains controversial..Although guidelines have mentioned that CSF PCT and lactate may serve as as biomarkers of intracranial infection after neurosurgery, they are not clearly recommended [16]. Therefore, the suitability of PCT as a potential indicator for diagnosing intracranial infection after neurosurgery remains a topic of debate.The main objective of this study was to investigate the influencing factors of CSF PCT and its clinical value.

## Materials and methods

### Study design and subjects

This study was conducted in accordance with the Declaration of Helsinki (2000) of the World Medical Association. This study was performed in the ICU of People's Hospital of Ningbo University between February 2021 and August 2022 and was approved by the Ethics Committee of the Affiliated People's Hospital of Ningbo University [2021(006)].CSF and serum samples were collected from all ICU neurosurgery patients with high fever (T > 38.5 °C) who were suspected to have intracranial infection. Patients diagnosed with other nonintracranial infections (including pneumonia, urinary tract infection, bloodstream infection, tuberculosis, syphilis), renal failure, immunosuppressant use, and confirmed intracranial infections were excluded from this study. Patients under the age of 18, pregnant women, patients with immune-mediated encephalitis, and patients with metabolic encephalopathy were also not eligible.

### Data collection and definitions

In this study, we considered elevated CSF PCT levels to be those exceeding the normal reference value (0.05 ng/ml). All participants were divided into the intracranial infection groupor nonintracranial infection group (referred to as the case–control group in subsequent text).

The diagnostic criteria for intracranial infection were as follows: (1) clinical manifestations such as postoperative fever, headache, neck stiffness, or change in consciousness; (2) glucose level of CSF < 2.5 mmol/L or a CSF/serum glucose ratio < 0.4; (3) a CSF white blood cell (WBC) count ≥ 1000 cells/µl and a polykaryocyte percentage ≥ 75%; and (4) positive CSF culture. The diagnosis of intracranial infection was made individually for patients meeting the fourth criterion. Intracranial infection was also diagnosed in patients with negative CSF culture results who met the other three diagnostic criteria.

PCT detection method: CSF specimens were acquired by lumbar puncture for all patients. Protein, chlorine, glucose and lactate levels were determined using an automated chemical analyzer (Diamond Diagnostics, Citrus Heights, CA, USA). PCT was detected by enzyme-linked fluorescence (Marcy L’Etoile, France). The lower limit of positivity was 0.05 ng/ml. Peripheral venipuncture blood was collected under aseptic conditions no later than 30 min after CSF collection.

### Statistical analysis

Data were analyzed using SPSS 25.0 statistical software (IBM, USA). Since the data of all parameters showed a skewed distribution (Kolmogorov–Smirnov, *p* < 0.05), continuous data were expressed as the median, and nonparametric tests were used for comparisons between two groups, while counting data were compared by chi-square tests. Factors with a *p* value < 0.2 on univariate analysis were further analyzed by multivariate logistic regression for influencing CSF PCT. ROC curve analysis was used to analyze the diagnostic efficacy. *P* < 0.05 was considered statistically significant. The figures were drawn with GraphPad Prism, version 6.01 (GraphPad, San Diego, CA, USA).

## Results

A total of 183 CSF samples were collected. There were 76 patients in the intracranial infection group, 30 males and 46 females, aged 38–81 years, with an average age of 62.61 ± 13.12 years, and 107 patients in the case‒control group, 49 males and 58 females, aged 42–85 years, with an average age of 62.83 ± 12.79 years. There were no statistically significant differences in age or sex between the two groups (*p* > 0.05). Among the collected samples, a total of148 were identified as having increased CSF PCT levels, including 73 in the intracranial infection group and 75in the case‒control group (Tables 1 and 2).

**Table 1:** Comparison of general data between intracranial infection group and case–control group

Factor	Intracranial infection group (*n* = 76)	Non-intracerebral infection group (107)	Z/T	P
Age(year)	62.62 ± 13.12	62.83 ± 12.79	0.773	0.059
Sex(male)	30(39%)	49(46%)	3.516	0.068
increased CSF PCT	73(96%)	75(70%)	19.360	0.001

There was no significant difference between the two groups except the increase rate of PCT in cerebrospinal fluid

**Table 2:** General information about the subject

Type of disease	Number of cases	Surgical method	Intracranial infections	Increased cerebrospinal fluid PCT
Parenchymal hemorrhage	54	Craniotomy hematoma removal	28 (52%)	46 (85%)
Subarachnoid Hemorrhage	24	Craniotomy aneurysm clipping/interventional embolization	12 (50%)	22 (92%)
Cerebral infarction	22	Decompressive craniectomy	10 (45%)	20 (91%)
Trauma	21	Brain debridement /Subdural (epidural) hematoma removal	9 (43%)	16 (76%)
Ventricular hemorrhage	18	Extra ventficular drainage	10 (56%)	17 (94%)
Hydrocephalus	15	Ventriculoperitoneal shunt	2 (13%)	8 (47%)
Skull loss	10	Cranioplasty	1 (10)	7 (70)
Cerebrospinal fluid Leakage	8	Surgical repair of cerebrospinal fluid leakage	1 (13%)	3 (38%)
Brain metastatic tumor	4	Craniotomy tumor resection	1 (25%)	4 (100%)
Pituitary tumors	3	Pituitary tumor resection	1 (33%)	2 (67%)
Meningiomas	3	Meningeal tumor resection	1 (33%)	3 (100%)
Brain arteriovenous malformations	1	Cerebrovascular bypass surgery	0 (0)	0 (0)

### Multivariate analysis of abnormal PCT in CSF after neurosurgery

Univariate analysis indicated statistically significant differences in the levels of CSF PCT among patients with intracranial infection and hemorrhagic CSF(including intraventricular hemorrhage, subarachnoid hemorrhage) groups.Howere, there were no significant differences in age, gender, Glasgow Coma Scale (GCS) score, epilepsy, serum and tumor (Table 3). Multivariate logistic regression analysis was performed on factors with *p* < 0.2, which demonstrated that patients with intracranial infection were more likely to have increased CSF PCT than patients without intracranial infection [OR = 0.117, 95% CI: 0.025–0.559; *p* < 0.007]. Patients with hemorrhagic CSF had higher CSF PCT than patients with nonhemorrhagic CSF [OR = 0.162, 95% CI: 0.029–0.916; *p* < 0.039] (Table 4).

**Table 3:** Univariate Analysis results of PCT increase in cerebrospinal fluid (CSF) after neurosurgery

Factor	PCT abnormal group (*n *= 35)	PCT increase group (*n* = 148)	Z/T	P
Age(year)	66.45 ± 15.22	59.83 ± 15.93	0.773	0.09
Sex(male)	15(43%)	79(53%)	1.708	0.34
GCS	6(5 ~ 6)	6(4 ~ 7)	-1.01	0.92
Trauma	5 (14.3%)	16 (11%)	0.336	0.56
Tumor	2 (6%)	9 (6%)	3.037	0.91
Epilepsy	0	12 (8%)	2.696	0.12
Intracranial infection	3 (9%)	73 (49%)	19.3	0.01
Hemorrhagic CSF	3 (9%)	39 (26%)	5.06	0.03
Foreign body implantation	9(26%)	22(15%)	1.54	0.12
Serum PCT	0.13(0.06 ~ 0.27)	0.17(0.08 ~ 0.49)	0.034	0.29

Univariate analysis showed that intracranial infection and Hemorrhagic CSF had statistical differences between the two groups

**Table 4:** Multivariate analysis results of PCT increase in CSF after neurosurgery

Factor	b	SE	wald	P	OR	95%CI
Age(year)	0.02	0.02	0.003	0.32	1.02	0.98–1.52
Trauma	1.23	0.78	2.49	0.12	3.43	0.76–15.45
Epilepsy	-19.45	10,573.14	0	0.99	0.00	0.00-
Intracranial infection	-2.14	0.78	7.235	0.01	0.12	0.03–0.56
Hemorrhagic CSF	-1.82	0.88	4.24	0.04	0.04	0.03–0.91
GCS	0.28	1.29	1.98	0.83	1.03	0.78–1.32
Foreign body implantation	1.11	0.61	3.39	0.63	3.02	0.93–9.83

Multivariate analysis of logistics suggested that intracranial infection and hemorrhagic CSF were independent risk factors for increased CSF PCT after neurosurgery

### Comparison of CSF and serum PCT between the intracranial infection group and the case‒control group

**Fig. 1:**
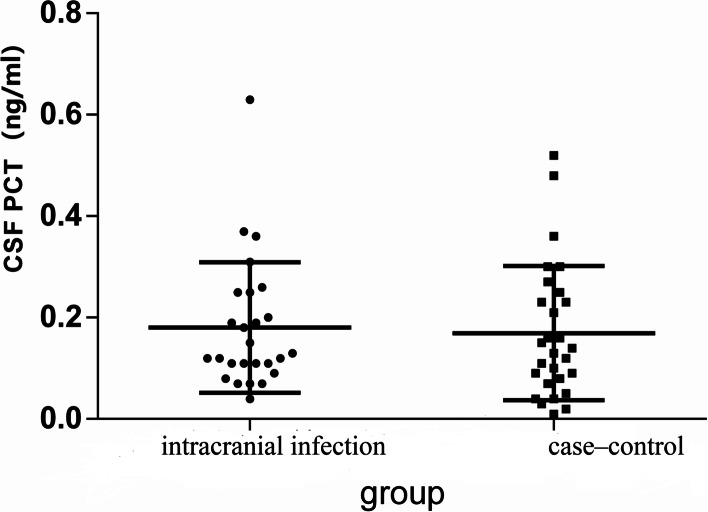
There was no statistically significant difference in CSF procalcitonin levels between the intracranial infection group and the case‒control group (Mann‒Whitney, *P* > 0.05)

**Fig. 2:**
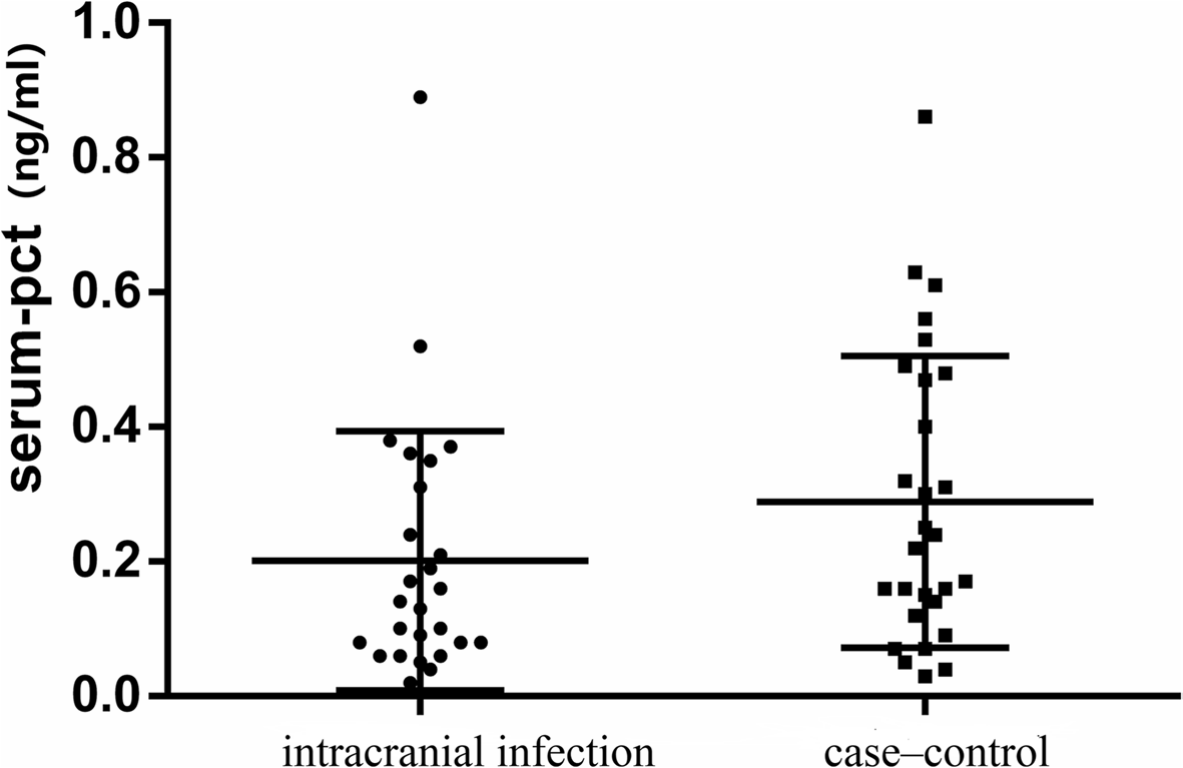
There was no statistically significant difference in serum procalcitonin levels between the intracranial infection group and the case‒control group (Mann‒Whitney, *P* > 0.05)

**Fig. 3:**
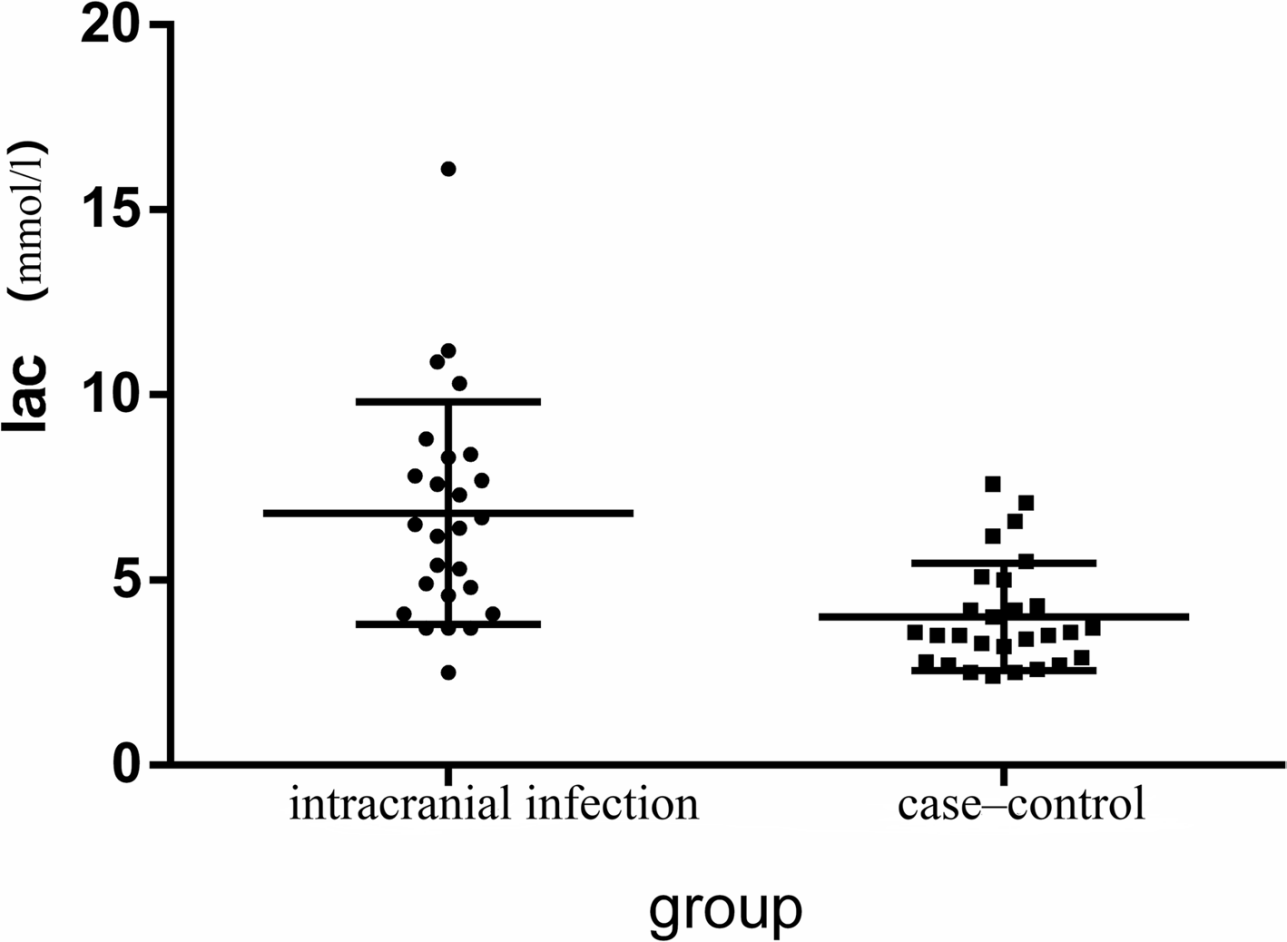
There was a significant difference in CSF lactate between the intracranial infection group and the case‒control group (Mann‒Whitney, *P* > 0.05)

**Fig. 4:**
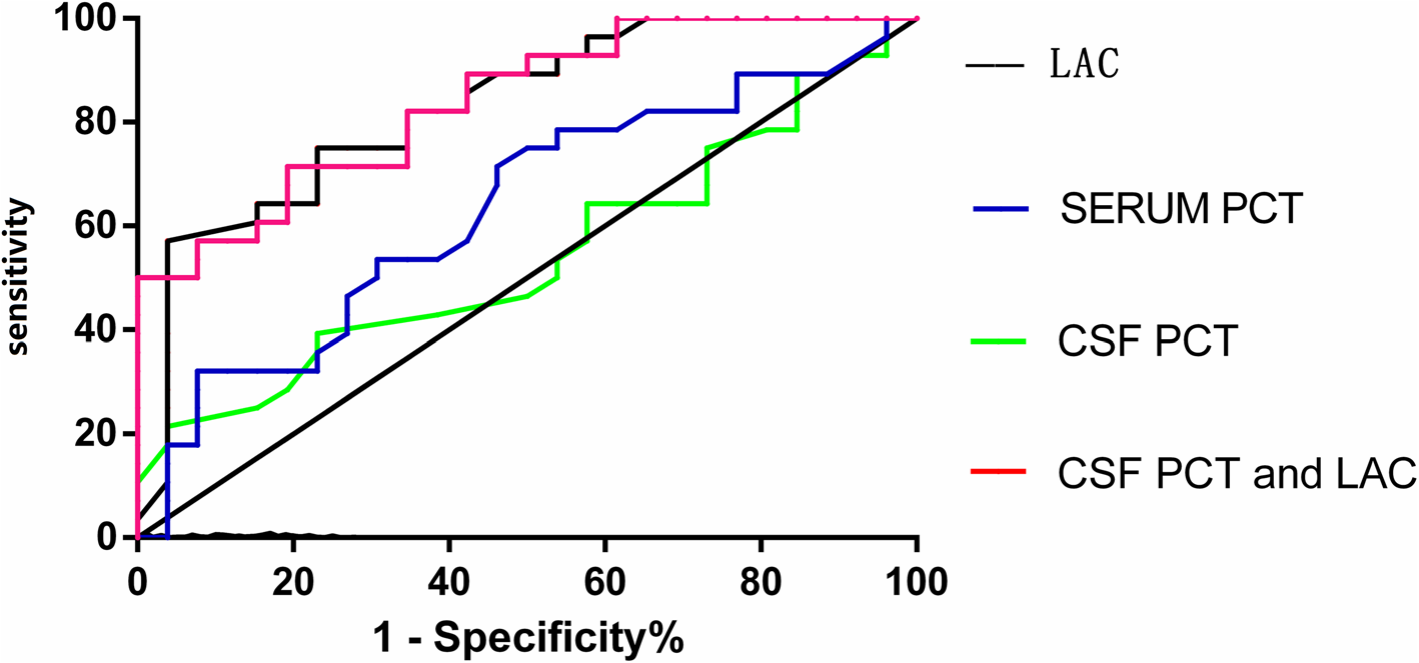
The ROC curve suggested that CSF lactate and CSF PCT combined with lactate had better diagnostic efficacy, but CSF PCT or serum PCT had poor diagnostic efficacy

The median CSF PCT levels in the intracranial infection group and the case‒control group were 0.13 (0.11, 0.25) ng/ml and 0.14 (0.07, 0.25) ng/ml, respectively, with no significant difference between the two groups (*p* > 0.05, Fig. 1). The median serum PCT levels in the intracranial infection group and the case‒control groupwere 0.14 (0.08,0.32) ng/mland 0.23 (0.13,0.48) ng/ml, respectively, with no significant difference between the two groups (*p* > 0.05, Fig. 2). The median values of CSF lactate in the intracranial infection group and the case‒control group were 6.45 (4.475, 8.325) mmol/l and 3.2 (2.02, 4.200) mmol/l, respectively, and the difference between groups was statistically significant (*p* < 0.05, Fig. 3). The areas under the ROC of CSF PCT, serum PCT,CSF lactate, andCSF PCT combined with lactate were 0.59, 0.63, 0.82, and0.83,respectively (*p* > 0.05, Fig. 4).

## Discussion

Studies have provided evidence that PCT in CSF increases in in cases of intracranial infection following neurosurgery**.** However, there are conflicting results regarding whether this increase is due to the secretion of brain tissue or the elevated serum PCT levels after breaching the blood–brain barrier. The potential use of CSF PCT in diagnosing intracranial infection after neurosurgery remains a topic of debate. Additionally, it has been observed that some patients without intracranial infection also exhibit increased CSF PCT levels, suggesting that the elevation of CSF PCT may not be specific to infection.

This particular study discovered that not only did 73 patients with intracranial infection exhibit increased CSF PCT levels, but 75 patients without intracranial infection also showed similar elevations.These finding suggesting that the increase in CSF PCT may not be specific to infection and is instead nonspecific. Furthermore, when performing multivariate analysis, it was revealed that hemorrhagic CSF could also impact CSF PCT results. This finding aligns with a previous study by Wang et al. that reported increased PCT in the CSF of patients with subarachnoid hemorrhage [15]. However, the degree and mechanism of CSF PCT elevation caused are still unclear.Intracranial infection is an independent risk factor for the elevation of procalcitonin in CSF. However,this study did not find significant difference in PCT between the intracranial infection group and the aseptic meningitis group. It is important to note that our analysis is influenced by the presence of several confounding factors, particularly the uneven distribution of patients with hemorrhagic CSF in both groups.. Additionally, a study by Zhang et al. [17] revealed that CSF PCT was correlated with BBB permeability and serum PCT, which seems to suggest that CSF PCT is derived from serum. Therefore, further research is needed to better understand the role and interpretation of CSF PCT in differentiating intracranial infections from other conditions. Indeed, a study by Radant et al. [18] demonstrated the presence of PCT in the medium of trigeminal glial cells, which supported that brain tissue could also produce and secrete PCT in addition to serum. This finding helps explain the limited and controversial clinical value of using elevated CSF PCT as a sole diagnostic marker for intracranial infection following neurosurgery. It implies that CSF PCT levels may be influenced by factors other than direct CNS infection, such as blood–brain barrier permeability or systemic inflammation. Therefore, further research is needed to better understand the precise origin and interpretation of CSF PCT in the context of intracranial infections.Of course, some studies have also found that the diagnostic efficacy can be improved by the combined examination of CSF PCT and other indicators. For example, Zheng et al.concluded that the combined detection of CSF PCT, CSF lactate, CSF protein concentration and CSF blood glucose ratio has good diagnostic value for meningitis after neurosurgery, which can shorten the diagnostic time of patients and improve the success rate of patients' treatment [19]. During intracranial bacterial infection, the number of bacteria in CSF is very large, and a large number of white blood cells enter the CSF to phagocytose bacteria. The oxygen demand increases sharply, the oxygen content of CSF decreases, and the production of lactate increases.Lactate in CSF is a byproduct of bacterial metabolism that is not affected by lactate in the blood. Therefore, a rapid increase in lactate levels in CSF in a short time can help effectively diagnose craniocerebral infection [20]. In Kristian's study, cerebrospinal fluid lactate levels greater than 3.5mmom/l were found to distinguish between acute meningitis and aseptic meningitis [21]. In this study, we also found a statistical difference in lactate between the two groups, and we also found that if CSF PCT combined with lactate was used to diagnose intracranial infection, the area under the ROC curve was large, which could significantly improve the diagnostic efficacy of intracranial infection after neurosurgery.

The finding of this study may be biased due to the absence of multicenter support and the relatively small number of cases. The analysis of the specific pathogens causing intracranial infections was limited due to the small number of samples available. Furthermore, the correlation between the degree of hemorrhagic CSF and the level of PCT in CSF was not investigated in this study. Furthermore, this study did not investigate the source of CSF PCT, which is not conducive to the diagnostic value of CSF PCT for intracranial infection after neurosurgery.

## Conclusions

The presence of elevated PCT in the CSF of patients with high fever after neurosurgery is nonspecific and can be influenced by factors other than intracranial infection. Hemorrhagic CSF is an independent risk factor for increased CSF PCT. Therefore, relying solely on CSF PCT for diagnosing intracranial infections has limited diagnostic value. However, combining CSF PCT with other indicators may improve its diagnostic efficacy. It is important to note that the small sample size of this study warrants further investigation with larger samples and a multicenter approach.

To gain a better understanding of the mechanism of CSF PCT changes during intracranial infections, we plan to establish a rabbit model of bacterial ventriculitis using different Gram staining types. This will allow us to explore the specific role of CSF PCT in the context of different types of bacterial infections. By conducting this study, we hope to enhance our understanding of CSF PCT as a diagnostic marker and contribute to the development of more accurate diagnostic tools for intracranial infections after neurosurgery.

## Data Availability

Data were collected from the Department of Intensive Care Unit of the Affiliated People's Hospital of Ningbo University. Since this research project has not been fully concluded, the data pertaining to the results of this research are not suitable for disclosure. Datasets used and/or analyzed in the current study can be obtained from the corresponding author on reasonable request. Email: whj269696@163.com.

## Abbreviations

CSF: Cerebrospinal fluid
CNS: Central nervous system
PCT: Procalcitonin
WBC: White blood cell
BBB: Blood–brain barrier
